# Chinese STEM College Students’ AI-Mediated Informal Digital Learning of English: A Hybrid SEM-PNA Approach to the Hedonic-Motivation System Adoption Model

**DOI:** 10.3390/jintelligence14070120

**Published:** 2026-06-25

**Authors:** Yixuan Xu, Hanwei Wu

**Affiliations:** 1 National Research Centre for Foreign Language Education, Beijing Foreign Studies University, Beijing 100089, China; 202420213014@bfsu.edu.cn; 2 Foreign Studies College, Hunan Normal University, Changsha 410081, China

**Keywords:** AI-mediated IDLE, STEM college students, hedonic motivation, PLS-SEM, PNA

## Abstract

English proficiency is vital for non-native speakers’ career development, yet classroom instruction alone cannot meet practical demands, making informal digital learning of English (IDLE) increasingly important. Artificial intelligence (AI), with conversational and multimodal functions, offers new opportunities for IDLE. However, existing research on AI-mediated IDLE has predominantly focused on language majors and often relied on a single methodological lens, neglecting STEM undergraduates and the complex network dynamics among motivational factors. However, research has largely focused on language majors, leaving STEM majors underexplored. Guided by the Hedonic-Motivation System Adoption Model (HMSAM), this study analyzed data from 413 Chinese STEM majors using partial least squares structural equation modeling (PLS-SEM, SmartPLS 4.0) and psychological network analysis (PNA, R 4.5.3). PLS-SEM results showed that enjoyment was the strongest direct predictor of AI-IDLE, followed by focused immersion, perceived usefulness, and curiosity. Control contributed indirectly via focused immersion, while boredom was non-significant. Perceived ease of use influenced AI-IDLE only through cognitive and emotional pathways. The model explained 58.1% of the variance. PNA further identified enjoyment, focused immersion, and control as central nodes, while the link between perceived usefulness and AI-IDLE was non-significant. These findings suggest that Chinese STEM undergraduates’ AI-IDLE is primarily driven by intrinsic hedonic motivations rather than utilitarian evaluations. The study provides empirical support for designing AI tools that enhance enjoyment and control to foster STEM students’ extracurricular English engagement.

## 1. Introduction

English proficiency as a foreign language (EFL) has become a critical determinant of career development and salary prospects in globalized professional environments. Research consistently shows a positive association between English proficiency and employment opportunities, career advancement, and income levels (Peltokorpi, 2023; Prasetya, 2023; Rana & Shaikh, 2024). In high-tech industries, multinational corporations, and research contexts, strong English skills are indispensable for securing competitive positions and higher remuneration. For non-native learners, however, classroom-based instruction alone rarely suffices to achieve fluency (G. Liu et al., 2026). Classroom settings often provide limited input, restricted interaction, and narrow communicative contexts, which hinder progress. Consequently, extracurricular learning has become essential for supplementing and strengthening language competence (G. L. Liu et al., 2025; Zhao et al., 2026). Informal English learning, defined as learner-initiated practices outside formal education, has attracted growing scholarly attention for its authentic contexts, richer input, and flexible pacing (Soyoof et al., 2026).

The rapid advancement of digital technologies has created new opportunities for informal English learning. Through the internet, mobile devices, and social media, learners can access multimodal content such as videos, podcasts, games, and social platforms, which provide authentic input and natural contexts (Reinhardt, 2019). With the rise of artificial intelligence (AI), language learning has entered a transformative stage. Tools like ChatGPT-4, built on large language models, can simulate human conversation and support voice interaction, aligning with the core requirements of language learning: authentic, comprehensible, and interactive practice (Wu et al., 2026; Wu & Pan, 2025; Zhao & Wang, 2026). Compared with traditional digital resources, AI offers instant feedback, personalized dialogue, and controllable practice environments, making it particularly effective in advancing informal digital English learning (IDLE) (G. Liu & Ma, 2023; G. Liu et al., 2023; Soyoof & Reynolds, 2026).

Recent empirical studies have provided compelling evidence for the effectiveness of AI-IDLE. For instance, Aladini et al. (2025) demonstrated that AI-supported autonomous writing tasks significantly enhanced learners’ writing proficiency and grammatical accuracy, while Chen et al. (2025) reported that AI-based speaking training improved the oral competence of Chinese university students. Together, these findings suggest that AI can play a constructive role in informal learning contexts. Nevertheless, as Wu et al. (2024) have emphasized, the impact of technology ultimately depends on learners’ actual engagement. In other words, the potential of AI to facilitate English learning is contingent upon learners’ willingness to adopt and practice AI-IDLE outside the classroom. Consequently, examining the factors that shape learners’ AI-IDLE behavior has become a central focus of current research (Soyoof et al., 2025).

Several studies have investigated the factors influencing AI-IDLE, highlighting the roles of emotional experiences such as enjoyment, curiosity, and boredom, along with learning motivation, self-efficacy, digital competence, and social support (G. Liu & Hossain, 2026; G. Liu & Zhao, 2025b; Qu & Wu, 2024; Zhao & Danping, 2024). Yet most of these studies have concentrated on learners with immediate and explicit demands for English, including language majors, students preparing for overseas study, and international students already enrolled in English-speaking contexts. These groups typically face clear pressures from examinations, academic writing requirements, or daily communication needs, which make their motivation to use AI both stronger and more visible. By contrast, STEM (science, technology, engineering, and mathematics) undergraduates have received far less attention, leaving a critical gap in the current literature.

STEM undergraduates account for a large share of global higher education, and their English learning needs are distinctive. Although not language majors, they rely on English for research reading, international conferences, technical documentation, academic writing, and future employment in multinational enterprises (Montgomery, 2013). From the perspective of English for specific purposes, language majors focus on general communicative competence, whereas STEM students need discipline-specific academic English (Xie, 2019). Unlike language students, their demand for English is typically long-term and context-triggered rather than exam-driven, making their motivation more intrinsic and their use of AI potentially different (Ye, 2020). Heavy coursework and laboratory tasks further limit time for dedicated English study, rendering fragmented, low-cognitive-load, and entertainment-oriented AI-IDLE a practical pathway for improving proficiency (M. Wang & Zhang, 2026). Understanding the factors shaping AI-IDLE among STEM students not only addresses a critical research gap but also informs the design of educational technologies and targeted curricular support.

Building on this background and given that prior research on AI-IDLE has largely relied on single-method analyses that cannot distinguish net effects from unique pairwise associations, the present study employs the Hedonic Motivation System Adoption Model (HMSAM) as its theoretical framework and integrates partial least squares structural equation modeling (PLS-SEM) with psychological network analysis (PNA) to investigate the factors shaping AI-IDLE among Chinese STEM undergraduates. Specifically, it examines the path relationships between HMSAM constructs including perceived ease of use, perceived usefulness, enjoyment, curiosity, control, focused immersion, and boredom, and AI-IDLE, while identifying the most influential nodes within the psychological network. This study seeks to advance understanding of technology-enabled language learning among non-language majors and to provide empirical evidence and practical guidance for supporting extracurricular English learning in STEM contexts.

## 2. Literature Review

### 2.1. Informal Digital Learning of English Mediated by AI

The concept of IDLE has undergone a gradual evolution from “informal learning” to “digital language learning” and ultimately to “out-of-class English exposure.” Its theoretical foundation lies in informal learning research (Livingstone, 2001), which emphasizes learner-initiated activities outside formal education, where goals, content, and pace are autonomously determined. With the expansion of the internet, mobile devices, and social media, scholars began to examine learners’ extensive English exposure in everyday digital environments. Early notions such as “out-of-class learning” and “extramural English” highlighted authentic input, natural contexts, and spontaneous use beyond the classroom (Reinders & Benson, 2017; Sundqvist & Sylvén, 2016). The rise of Web 2.0 further underscored the interactive and participatory nature of digital platforms, prompting renewed attention to their role in language learning (Reinhardt, 2019). Building on this trajectory, Lee (2019) formally introduced the concept of IDLE, identifying its defining features as digital mediation, informality, autonomy, and learner-driven practices. Importantly, IDLE encompasses a continuum of behaviors ranging from entertainment-driven exposure to deliberate practice with explicit learning goals. As research has advanced, IDLE has been recognized as a complementary learning ecology that supplements and extends classroom instruction. A recent meta-analysis by G. L. Liu and Soyoof (2026) confirmed that IDLE exerts a moderate yet significant effect on English proficiency, thereby consolidating its role as an important dimension of contemporary language learning.

AI has emerged as a powerful medium within IDLE, offering personalized, immediate, and highly controllable language practice that aligns closely with IDLE’s autonomy and fragmented learning patterns (X. Wang et al., 2025; Wu & Wang, 2025). Recent studies demonstrate that AI-IDLE enhances multiple aspects of English proficiency, including reading, writing, and speaking (G. Liu & Zhao, 2025a), with particularly strong evidence in writing and speaking. In writing, Aladini et al. (2025) conducted a quasi-experimental study with 561 intermediate EFL learners in Iran, showing that AI-supported autonomous writing tasks significantly improved writing proficiency and grammatical accuracy compared with traditional instruction. Similarly, Zhang et al. (2025) developed a ChatGPT-based environment for 40 second-language learners, finding that three weeks of intervention effectively enhanced logical reasoning in argumentative writing. In speaking, Chen et al. (2025) implemented an eight-week AI-based speaking program with 71 Chinese undergraduates, reporting improvements across proficiency levels, with vocabulary gains more pronounced among advanced learners and fluency gains more evident among lower-level learners. Likewise, Zou et al. (2024), based on a survey of 366 undergraduates, found that AI tools significantly improved oral skills, including pronunciation, grammatical accuracy, reading aloud, and presentation ability. Collectively, these findings highlight the positive impact of AI-IDLE on English proficiency, justifying our examination of the factors that influence Chinese STEM undergraduates’ engagement in such learning behaviors.

### 2.2. Influencing Factors of AI-Mediated IDLE

Scholars have increasingly examined learners’ AI-IDLE and the factors shaping such behavior. Drawing on the HMSAM, Qu and Wu (2024) emphasized the direct role of emotional experiences in driving AI-IDLE. Their study showed that Chinese international students’ enjoyment, immersion, curiosity, and reduced boredom when using tools such as ChatGPT significantly predicted engagement, suggesting that immediate emotional states explain this behavior more effectively than instrumental utility. Beyond hedonic motivation, traditional learning motives also remain important. G. Liu and Hossain (2026), in a study of rural Bangladeshi undergraduates, found that self-efficacy, enjoyment, and the ideal L2 self positively predicted both IDLE and AI-IDLE, with prior IDLE experience emerging as the strongest antecedent, indicating that AI-IDLE is deeply embedded in learners’ existing digital learning habits. From a socio-developmental perspective, G. Liu et al. (2026) identified a chain pathway from social support to resilience, which then accumulates through receptive and productive IDLE before leading to AI-IDLE, underscoring its progressive and developmental nature. Meanwhile, individual competence factors also impose constraints. G. Liu and Zhao (2025b) reported that Chinese university EFL learners’ confidence, digital competence, and access to devices significantly predicted AI-IDLE, whereas demographic variables such as ethnicity had limited impact. This suggests that, given equal access to technology, effective engagement depends more on learners’ digital literacy and language confidence than on broader social identity.

Taken together, existing studies highlight four major dimensions influencing AI- IDLE: immediate emotional experiences, learning motivation and prior behavioral habits, individual competence, and social support with developmental pathways. Yet most research has focused on language majors, Global South populations, or international students, with insufficient attention to non-language majors, particularly STEM undergraduates whose IDLE demand is relatively weaker. This gap represents a critical limitation in the current literature.

### 2.3. Hedonic-Motivation System Adoption Model as the Basis

As noted earlier, existing research has devoted limited attention to non-language majors, particularly STEM undergraduates. Although these students face less immediate external pressure to learn English, they nevertheless encounter authentic contexts of use in research reading, technical documentation, and future employment. Their AI-IDLE behaviors may therefore differ from those of language majors. Among current technology acceptance models, the Hedonic-Motivation System Adoption Model (HMSAM) appears more suitable than the Technology Acceptance Model (TAM) or the Unified Theory of Acceptance and Use of Technology (UTAUT) for explaining AI-IDLE among Chinese STEM students (Lowry et al., 2013). TAM (Davis, 1989) and UTAUT (Venkatesh et al., 2003) emphasize perceived usefulness and ease of use, focusing on extrinsic motivation. Yet STEM students’ instrumental need for English is less urgent, making such external drivers less compelling. HMSAM, while retaining instrumental constructs, incorporates enjoyment, curiosity, control, and immersion, thereby better capturing behaviors driven by intrinsic motivation. Consequently, HMSAM may provide stronger explanatory power for AI-IDLE in STEM contexts.

**Figure 1:**
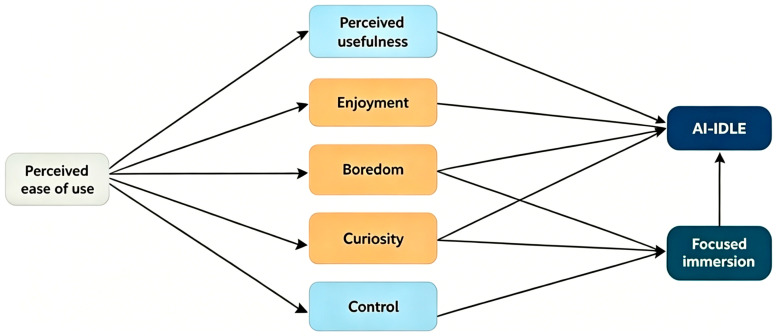
The HMSAM comprising boredom and AI-IDLE.

HMSAM also specifies clear relational paths among its constructs. Perceived ease of use positively influences perceived usefulness, curiosity, enjoyment, and control. Curiosity and enjoyment directly enhance usage intention and simultaneously strengthen focused immersion, while control directly increases focused immersion. Focused immersion, in turn, positively affects usage intention. This study further introduces boredom as a key variable, defined here as the boredom experienced during AI-IDLE activities rather than general boredom in English learning (see Figure 1). From the perspective of valence and activation, boredom is a negative deactivating emotion characterized by low arousal and limited cognitive engagement (Pekrun, 2006). In non-mandatory, self-directed informal learning contexts, the most common emotional challenge is not high-arousal anxiety but low-arousal boredom (Qu & Wu, 2024).

Finally, this study replaces behavioral intention in HMSAM with AI-IDLE as a concrete behavioral indicator. Behavioral intention, widely used as a dependent variable in TAM and HMSAM, reflects individuals’ tendencies to use a technology. However, intention and actual behavior may diverge, particularly for STEM undergraduates in non-mandatory contexts, where self-reported intentions may not correspond to real practices. AI- IDLE directly measures learners’ actual behaviors, such as engaging in English conversations with AI or using AI to revise writing (G. Liu et al., 2024). This substitution grounds the research more firmly in observable behavior and avoids the intention–behavior gap. Moreover, IDLE itself is inherently autonomous and unsupervised (Lee, 2019). Using AI-IDLE as the outcome variable thus more directly reflects the driving effects of HMSAM antecedents on actual learning practices.

### 2.4. The Present Study: Combining PLS-SEM and PNA

From a methodological standpoint, PLS-SEM and PNA offer complementary strengths in multivariate research. PLS-SEM is a prediction- and explanation-oriented approach that estimates structural relationships among latent variables while assessing overall model fit and explanatory power (Hair et al., 2020). It requires minimal distributional assumptions, works well with relatively small samples, accommodates complex model structures, and emphasizes maximizing the explained variance (R^2^) of endogenous variables. Thus, PLS-SEM is particularly effective for testing whether a theoretical model holds and whether hypothesized paths among latent constructs are statistically significant.

Nevertheless, SEM approaches rely on latent variable construction and linear assumptions, which limit their capacity to capture direct and fine-grained associations among observed variables. PNA offers an alternative analytic logic by conceptualizing variables as interconnected nodes and estimating edge weights to represent direct relationships without latent constructs or predefined path structures (Hevey, 2018). This approach enables the identification of central variables, bridging variables, and potential relational clusters, thereby revealing the internal dynamics and structural characteristics of the system.

Integrating PLS-SEM and PNA allows researchers to obtain complementary insights from distinct analytic perspectives. PLS-SEM emphasizes the testing of theoretical structures and latent paths, whereas PNA highlights direct associations among observed variables and network topology. Together, they enhance robustness and mitigate the limitations of relying on a single method. For instance, a variable may show a nonsignificant path in PLS-SEM yet exhibit high centrality in the network, indicating its critical role in system dynamics, or vice versa. Consequently, recent studies, such as Paradowski and Jelińska (2024) and X. Wang et al. (2026), have increasingly combined both approaches to achieve a more comprehensive understanding of complex psychological and behavioral systems in language learning.

Specifically, this study adopts the HMSAM as its theoretical foundation and employs a hybrid SEM-PNA approach to investigate the factors influencing AI-IDLE among Chinese STEM college students. More precisely, the study seeks to address the following two research questions:

**RQ1.**  How are the HMSAM constructs associated with AI-IDLE among Chinese STEM college students?
**RQ2.**  Which constructs demonstrate the highest influence within the psychological network comprising the HMSAM constructs and AI-IDLE?

## 3. Methodology

### 3.1. Participants

Using purposive sampling, we distributed questionnaires through our social networks via Wenjuanxing (an online platform for data collection) to students from three universities in Zhejiang and Hunan, China. The inclusion criteria were: (1) enrollment in a STEM major, and (2) prior experience using AI for IDLE. Initially, 518 responses met the inclusion criteria. However, 105 cases were excluded for the following reasons: (1) uniform responses across all items, and (2) incorrect answers to embedded attention-check questions. The final sample comprised 413 participants, representing STEM disciplines such as mathematics, statistics, physics, chemistry, biology, geography, computer science, electronic information engineering, and mechanical engineering. The original data could be seen in the Supplementary Materials. The detailed demographic characteristics of the participants are presented in Table 1.

### 3.2. Instruments

#### 3.2.1. Perceived Ease of Use and Perceived Usefulness

The items used to assess perceived ease of use (5 items) and perceived usefulness (5 items) were adapted from Wu et al. (2024), specifically designed for AI-IDLE. Each item was rated on a 7-point Likert scale. Higher total scores indicate stronger levels of perceived ease of use and perceived usefulness.

#### 3.2.2. Enjoyment and Boredom

The items used to assess enjoyment (4 items) and boredom (4 items) were adapted from Wu and Liu (2025), specifically developed for AI-IDLE. Each item was rated on a 7-point Likert scale, with higher total scores indicating stronger levels of enjoyment and boredom.

#### 3.2.3. Curiosity, Control and Focused Immersion

The items used to assess curiosity (3 items), control (3 items), and focused immersion (4 items) were adapted from Deng and Yu (2023). The original items were developed for TikTok-based learning contexts and were modified for AI-IDLE. Specifically, we replaced references to “TikTok” with “AI”, while keeping the original semantic structure and response format intact. For curiosity, an example item is *“Using AI for English learning stimulated my curiosity.”* For control, an example item is *“I had control over my interaction with AI.”* For focused immersion, an example item is *“I was engrossed in English learning with AI.”* Each item was rated on a 7-point Likert scale, with higher total scores indicating stronger levels of curiosity, control, and focused immersion.

#### 3.2.4. English Proficiency

English proficiency was included as a control variable and measured using a single self-reported item: *‘I believe my English proficiency is…’*, rated on a 5-point scale from 1 (very poor) to 5 (very good).

### 3.3. Data Analysis

This study employed PLS-SEM using Smart PLS 4, following the staged procedures recommended by Hair et al. (2020). The first step involved measurement model evaluation, where confirmatory composite analysis (CCA) was conducted. Key criteria included factor loadings, reliability indices, convergent validity, and discriminant validity, ensuring that each item accurately captured the intended construct. The second step focused on structural model assessment, in which bootstrapping was used to test the significance of path relationships. This procedure generated *p*-values and confidence intervals (CI) for path coefficients. The criteria for significance were as follows: direct paths were considered significant if *p* < 0.05, and indirect paths were deemed significant if the 95% confidence interval did not include zero.

PNA was conducted using R 4.5.3. First, a regularized partial correlation network was estimated by treating all constructs as nodes. The network was built under the Gaussian graphical model framework and regularized using LASSO with EBIC selection to reduce spurious edges and improve interpretability (Epskamp & Fried, 2018). We then generated 1000 non-parametric bootstrap samples to obtain 95% confidence intervals for edge weights, allowing evaluation of the accuracy of the estimated associations. To identify key variables, we computed node strength, closeness, betweenness, and expected influence, which together capture each node’s connectivity, influence, and structural importance within the network (Borsboom et al., 2021). Finally, network stability was assessed using case-dropping subset bootstrapping. We examined the correlation stability of edge weights and node strength, and judged robustness based on the recommended criterion that the correlation stability coefficient should be at least 0.5 (Epskamp et al., 2018).

## 4. Results

### 4.1. PLS-SEM Findings

To evaluate the measurement model, CCA was conducted. Following the recommendations of Hair et al. (2020), the indicator loadings and their significance were first examined. According to the criterion proposed by Hair et al. (2020), items with factor loadings greater than 0.708 should be retained. The results showed that item PE5 under the *perceived ease of use* construct had a loading of 0.631, which did not meet the threshold and was therefore removed. As presented in Table 2, the factor loadings of the remaining items for each latent variable were all above 0.708, satisfying the measurement standards.

In the second and third steps, the reliability of the measurement model was assessed. Following the recommendations of Hair et al. (2020), both Cronbach’s α and composite reliability (CR) were reported, with acceptable thresholds set at 0.70. As shown in Table 2, the Cronbach’s α for all constructs ranged from 0.839 to 0.934 (all exceeding 0.70), and the CR values ranged from 0.891 to 0.945 (all above 0.70), indicating satisfactory internal consistency across constructs.

The fourth step examined convergent validity, evaluated using the average variance extracted (AVE). According to Hair et al. (2020), AVE values should exceed 0.50. As presented in Table 2, the AVE values for all constructs ranged from 0.672 to 0.810, demonstrating adequate convergent validity of the measurement model.

The fifth step tested discriminant validity using the criterion proposed by Fornell and Larcker (1981), which requires that the square root of each construct’s AVE be greater than its correlations with all other constructs. As shown in Table 3, all constructs met this criterion, confirming satisfactory discriminant validity among the study variables.

**Figure 2:**
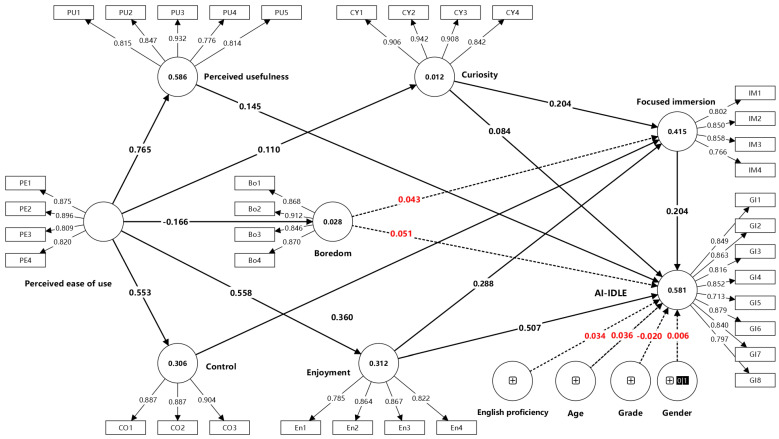
The structural model based on HMSAM (*n* = 413). *Note*: Red front = non-significant; Black front = significant.

The structural model was presented in Figure 2. The control variable estimates (see Table 4) indicate that age (β = 0.036, *p* = 0.610), gender (β = 0.006, *p* = 0.924), grade level (β = −0.020, *p* = 0.780), and English proficiency (β = 0.034, *p* = 0.297) all exerted non-significant direct effects on AI-IDLE. These findings suggest that neither demographic characteristics nor learners’ English proficiency had a meaningful influence on their engagement in AI-IDLE.

Among the key constructs (see Table 4), enjoyment demonstrated the strongest positive association with AI-IDLE (β = 0.507, *p* < 0.001), followed by perceived usefulness (β = 0.145, *p* = 0.021), curiosity (β = 0.084, *p* = 0.018), and focused immersion (β = 0.204, *p* < 0.001). Boredom, in contrast, was not significantly related to AI-IDLE (β = 0.051, *p* = 0.199).

For the antecedents of focused immersion, enjoyment (β = 0.288, *p* < 0.001), control (β = 0.360, *p* < 0.001), and curiosity (β = 0.204, *p* < 0.001) all showed significant positive associations, whereas boredom did not (β = 0.043, *p* = 0.306).

In addition, perceived ease of use was positively associated with perceived usefulness (β = 0.765, *p* < 0.001), enjoyment (β = 0.558, *p* < 0.001), control (β = 0.553, *p* < 0.001), and curiosity (β = 0.110, *p* = 0.031), and negatively associated with boredom (β = −0.166, *p* = 0.003).

The indirect effects are summarized in Table 5. The results show that perceived ease of use exhibited several significant indirect associations with AI-IDLE through different mediating pathways. First, the indirect pathway via perceived usefulness was significant (β = 0.110, 95% CI [0.017, 0.207]), indicating that perceived usefulness served as a meaningful mediator.

Among the emotional–motivational mediators, enjoyment demonstrated a strong and significant indirect association with AI-IDLE (β = 0.284, 95% CI [0.210, 0.357]). In addition, the sequential pathway perceived ease of use → enjoyment → focused immersion → AI-IDLE was also significant (β = 0.033, 95% CI [0.015, 0.055]). The pathway through control → focused immersion was similarly significant (β = 0.041, 95% CI [0.019, 0.066]).

In contrast, the indirect pathways involving boredom (either directly or sequentially through focused immersion) were non-significant, as were the indirect pathways involving curiosity (both the direct and sequential forms), given that their confidence intervals included zero. In addition, the model explained 58.1% variance of AI-IDLE in total.

### 4.2. PNA Findings

**Figure 3:**
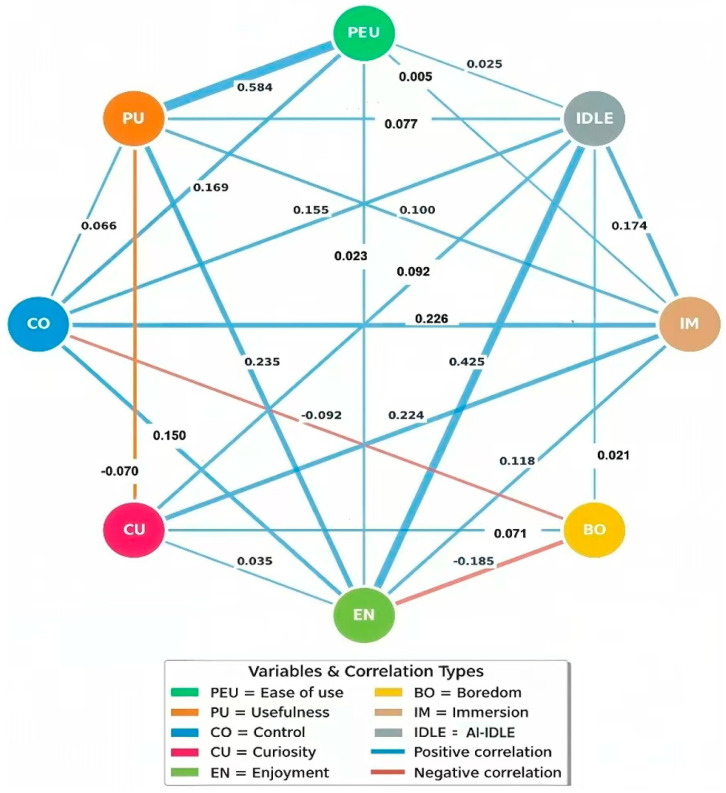
Structure of the estimated network (*n* = 413).

Using the EBICglasso estimation method (Epskamp et al., 2018), the psychological network comprised eight nodes with a theoretical maximum of 28 edges (see Figure 3). A total of 23 non-zero edges were retained, resulting in a network density of 0.821, indicative of a highly interconnected structure. Most edges were positive, with only two negative associations observed: EN–BO (−0.185) and CO–BO (−0.092), the former showing a stronger negative relationship.

The strongest edge weight was found between PEU–PU (0.584), followed by EN–IDLE (0.425), PU–EN (0.235), CO–IM (0.226), and CU–IM (0.224). For the focal outcome, informal digital English learning (IDLE), the key driving edges were EN–IDLE (0.425), IM–IDLE (0.174), and CO–IDLE (0.155), with the latter representing a moderate positive association. In contrast, PU–IDLE (0.077), PEU–IDLE (0.025), and BO–IDLE (0.021) showed very weak associations, suggesting limited direct relevance of perceived usefulness, perceived ease of use, and boredom to IDLE within the network.

Overall, enjoyment (EN), focused immersion (IM), and control (CO) emerged as the variables most strongly connected to informal digital English learning, with additional associations illustrated in Figure 3.

**Figure 4:**
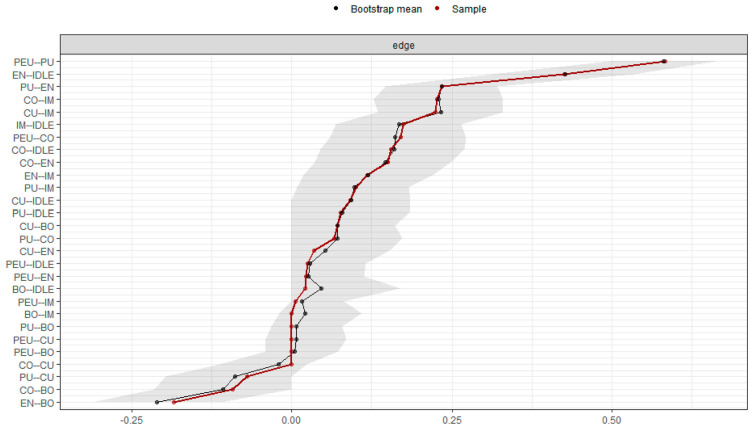
Accuracy of the estimated network (*n* = 413).

Figure 4 presents the accuracy of the estimated edge weights. Results from the non-parametric bootstrap procedure (1000 resamples) show that the confidence intervals for the key driving edges, EN–IDLE (95% CI = [0.317, 0.535]), IM–IDLE (95% CI = [0.066, 0.261]), CO–IDLE (95% CI = [0.043, 0.276]), and CU–IDLE (95% CI = [0.006, 0.179]), did not include zero, indicating that these associations are statistically reliable. In addition, the small discrepancies between the sample estimates (red lines) and the bootstrap means (black lines) further support the acceptable precision of the network structure.

In contrast, the confidence intervals for PU–IDLE (95% CI = [0.000, 0.181]), PEU–IDLE (95% CI = [0.000, 0.103]), and BO–IDLE (95% CI = [0.000, 0.159]) all included zero, suggesting that perceived usefulness, perceived ease of use, and boredom did not exhibit significant direct associations with IDLE in the network.

**Figure 5:**
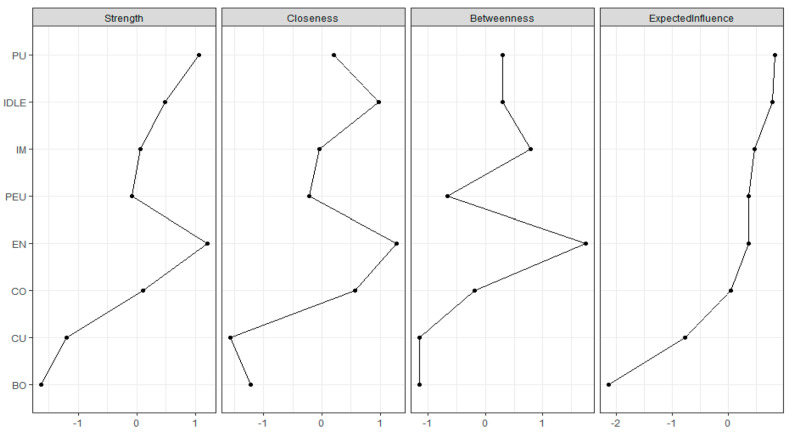
Centrality plot.

Figure 5 displays the relative positions of the eight variables across four centrality dimensions: strength, closeness, betweenness, and expected influence. EN had the highest betweenness centrality, serving as a key bridge connecting different subgroups in the network. PU stood out as the most dominant driver across strength, closeness, and expected influence. IM and IDLE were at medium-to-high levels on multiple indices, constituting secondary hubs. BO and CU scored the lowest on all centrality dimensions, positioning them at the periphery of the network.

**Figure 6:**
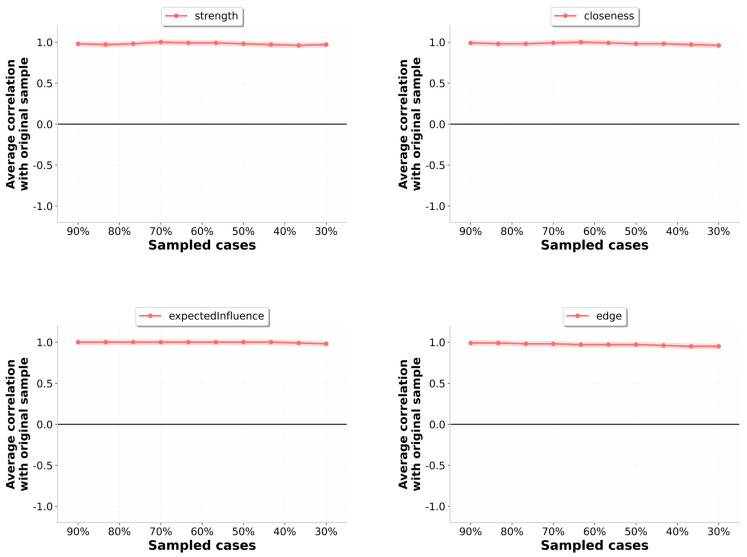
Stability of centrality indices and edge weights.

Network stability was assessed using the case-dropping bootstrap procedure (*n* = 500). As shown in Figure 6, the correlation stability (CS) coefficients for node strength, closeness, and expected influence, all exceeded 0.75, indicating that centrality rankings remained robust even when up to 75% of the sample was removed. Strength and expected influence showed the highest stability, while closeness displayed slightly greater variability but still fell within acceptable limits. In PNA, CS values above 0.50 are considered adequate; thus, the present results reflect excellent stability across all centrality metrics. Consistent with this, the average correlation of edge weights also surpassed 0.5 (Figure 6), underscoring the robustness of the estimated network.

## 5. Discussion

This study, grounded in the HMSAM and integrating PLS-SEM with PNA, systematically examined the factors influencing Chinese STEM college students’ engagement in AI-IDLE.

PLS-SEM results showed that enjoyment exerted the strongest direct positive effect on AI-IDLE, ranking highest among all direct paths to the outcome variable. Indirect effect testing further indicated that enjoyment also had a significant positive indirect influence through its enhancement of focused immersion. PNA provided structural corroboration: enjoyment displayed the highest node strength, reflecting the broadest and most intensive direct connections with other variables, and its edge weight with AI-IDLE was the strongest among all associations, underscoring its role as the most salient emotional antecedent. This dual validation is consistent with prior findings. Qu and Wu (2024), within the HMSAM, identified enjoyment as the core hedonic driver of Chinese international students’ use of ChatGPT, though its direct effect on behavioral intention was non-significant and operated mainly through focused immersion. Likewise, G. Liu and Hossain (2026) confirmed that enjoyment positively predicted AI-IDLE among Bangladeshi rural college students. The present study extends these insights by highlighting differences among STEM students: enjoyment functions both as a direct driver and as an indirect deepening mechanism. For international students, academic pressure and language adaptation needs mean that enjoyment must be transformed through focused immersion to influence behavior, whereas domestic STEM students, lacking strong external demands, engage in AI-IDLE as a self-initiated activity driven by intrinsic interest, allowing enjoyment to directly trigger learning behavior. This contrast suggests that the pathways of hedonic motivation within HMSAM are not fixed but vary across contexts and motivational hierarchies, reflecting differentiated transmission patterns.

In contrast to enjoyment, we found no significant effect of boredom on either focused immersion or AI-IDLE. Both the PLS-SEM path estimates and the edge-weight results from PNA indicated that boredom exerted only minimal influence. The network placed boredom at the periphery, with the lowest node strength and expected influence, and its edges with the outcome variable were weak. Boredom was negatively associated only with enjoyment and control, reflecting its inverse relationship with positive experiences but showing no direct role in driving learning behavior. This finding diverges from Qu and Wu (2024), who reported that boredom not only negatively predicted behavioral intention but also acted as a negative mediator between perceived usefulness and focused immersion. The discrepancy may be attributed to contextual differences: for international students, language learning is highly instrumental, and alleviating boredom becomes a motivational force; for STEM students, however, English learning involves little pressure, and their AI-IDLE is closely aligned with active exploration of novelty and enjoyment rather than passive avoidance of boredom (Ye, 2020). Moreover, the potential novelty effect of AI tools should be considered. At the time of data collection, many students were still in the early phase of using these tools, which were perceived as new. Under such conditions, boredom may not yet have had the chance to emerge. A cross-sectional design captures only this initial novelty-driven engagement rather than the satiated boredom that may develop with prolonged use. From the perspective of the bi-dimensionality of emotion, boredom represents a negative deactivating state with limited capacity to initiate new behaviors in autonomous contexts (Pekrun, 2006). The positive role of curiosity in this study further supports this interpretation: as a positive activating emotion, curiosity significantly predicted AI-IDLE in PLS-SEM and maintained stable connections with the outcome variable in the network, suggesting that STEM students’ learning is more strongly guided by exploratory desire than by the reduction in boredom.

The role of curiosity in STEM students’ AI-IDLE stands in marked contrast to prior findings. In the study by Qu and Wu (2024), curiosity showed no significant direct effects on either behavioral intention or focused immersion and was positioned as relatively peripheral. In our study, however, PLS-SEM revealed a significant direct effect of curiosity on AI-IDLE, although its indirect pathways were non-significant. PNA further demonstrated a stable direct edge between curiosity and the outcome variable, along with a robust connection to immersion, while its overall node strength remained modest, suggesting that curiosity operates in a specific rather than broad manner. Together, these results indicate that curiosity drives exploratory, immediate learning behaviors without requiring mediation through focused immersion. This finding engages in theoretical dialogue with studies of international students, where curiosity was non-significant, by showing that exploratory desire is a key driver among STEM students. Such distinctions underscore the need for more nuanced differentiation of hedonic motivations within HMSAM: enjoyment sustains and deepens engagement, whereas curiosity initiates exploratory attempts. Collectively, they represent the two central emotional forces shaping STEM students’ AI-IDLE.

The role of control is consistent with the assumptions of HMSAM. Qu and Wu (2024) reported that control significantly predicted focused immersion but had no direct effect on behavioral intention. Our study, drawing on both PLS-SEM and PNA, confirmed this pathway and revealed additional functions: control strongly predicted focused immersion, with the highest path coefficient, and was positively influenced by perceived ease of use. PNA further showed that control had the strongest edge weight with focused immersion and also maintained a direct link to AI-IDLE, ranking among the leading contributors. These findings suggest that in extracurricular learning, a sense of control is not only a prerequisite for immersion but can also directly stimulate usage behavior. This resonates with G. Liu et al. (2026), who emphasized that social support indirectly promotes AI-IDLE through resilience. Our study indicates that in self-directed contexts lacking external support, the control afforded by the technological environment may substitute for the “empowering” function of social support, enabling students to sustain learning behavior without supervision. Accordingly, the theoretical position of control within HMSAM should be reconsidered in light of learning autonomy: in highly self-directed informal learning, control serves not only as an antecedent of immersion but also as a direct driver of behavior.

One methodological nuance merits explicit attention. The direct effect of perceived usefulness on AI-IDLE was significant in PLS-SEM but non-significant in PNA, where the 95% CI crossed zero. This difference reflects the distinct analytical logics of the two methods rather than a contradiction. PLS-SEM estimates net effects while controlling for other constructs, which allows the modest influence of perceived usefulness to appear. PNA relies on regularized partial correlations that identify only the strongest unique pairwise associations after weaker links are penalized. In a dense hedonic dominated network where enjoyment, immersion, and control form the core motivational cluster, the incremental contribution becomes negligible once these stronger drivers are considered. This pattern offers a theoretically meaningful insight. For STEM students, utilitarian benefits are secondary and can be overshadowed by intrinsic motivations in a system-level structure. This nuance becomes visible only through a multi-method approach, which highlights how hedonic factors can dominate instrumental evaluations in shaping AI-IDLE and represents a distinctive contribution of this study.

At the level of individual competence, this study echoes the findings of G. Liu and Zhao (2025b), who identified confidence and digital competence as key predictors of AI-IDLE. Although these variables were not directly measured here, PLS-SEM results showed that perceived ease of use significantly predicted perceived usefulness, enjoyment, control, and curiosity, and exerted positive indirect effects on AI-IDLE through multiple pathways. PNA further revealed that perceived ease of use had non-significant edge weights with the outcome variable, but displayed the strongest edge with perceived usefulness. Together, these delineate a clear mechanism: perceived ease of use is not a direct behavioral driver but a foundational variable that indirectly shapes AI-IDLE by activating cognitive evaluations and emotional experiences. This finding is consistent with Qu and Wu (2024), who reported that perceived ease of use significantly predicted perceived usefulness, and aligns with the theoretical assumptions of HMSAM that its influence operates primarily through intrinsic motivational variables. Taken together, this study and G. Liu and Zhao (2025b) highlight the fundamental role of individual perception factors in driving AI-IDLE: while Liu and Zhao confirmed the predictive power of confidence and digital literacy, our study complements their work by demonstrating the indirect pathway of perceived ease of use as a foundational driver.

## 6. Implications and Limitations

This study has its theoretical contributions. This study demonstrates that the applicability of HMSAM varies across learner groups and contexts. While the model’s structural validity is confirmed in AI-IDLE in the previous study (Qu & Wu, 2024), the relative weight of motivational constructs differs: enjoyment and curiosity dominate in self-initiated STEM learning, whereas instrumental demands shape the pathways for international students. Moreover, the integration of PNA provides unique methodological value by revealing that enjoyment, immersion, and control form a densely interconnected core cluster, whereas perceived usefulness and boredom remain peripheral. This network structure, which PLS-SEM alone cannot detect, confirms that hedonic factors dominate not only in effect size but also in systemic centrality. These findings suggest that HMSAM should be applied with contextual sensitivity, recognizing differentiated motivational hierarchies across user populations.

The findings of this study provide implications for educational technology design, curriculum integration, and student support in STEM contexts. First, English learning support should prioritize hedonic-oriented AI interaction experiences rather than purely instrumental ones. AI modules can embed language practice into disciplinary scenarios such as scientific history narratives, virtual laboratory role-plays, or gamified academic debates, thereby fostering enjoyment and curiosity while situating English learning within authentic STEM environments. Second, strengthening learners’ sense of control is essential. AI tools should provide adjustable parameters, including speech rate, feedback detail, and task difficulty, and allow personalized settings to be saved. Such design reduces cognitive load and frustration, enabling students to sustain engagement in autonomous learning. Third, although boredom did not emerge as a significant factor in this study, its risk may increase with repeated use. Educators can mitigate this by refreshing task templates, introducing peer-challenge mechanisms, and incorporating multimodal resources such as videos, charts, and interactive simulations to maintain novelty. Finally, professional development for STEM instructors should emphasize the informal learning potential of AI rather than focusing solely on exam preparation. Teachers can encourage students to engage in low-stakes English practice outside the classroom, such as simulating international conference Q&A sessions or drafting laboratory logs, thereby embedding English into their academic toolkit rather than treating it as an additional burden.

This study has several limitations. First, the cross-sectional design does not permit causal inference. Although HMSAM presupposes directional pathways (e.g., enjoyment → immersion → behavior), the possibility of reverse causality cannot be excluded. For example, frequent AI-IDLE users may develop stronger feelings of control simply through familiarity with the tool. Future research could employ longitudinal designs or experience sampling methods (ESM) to examine dynamic causal relationships. Second, the sample was limited to STEM students from three universities in Zhejiang and Hunan, which constrains external validity. Expanding the sample size and including more diverse populations would strengthen generalizability. Third, reliance on self-report measures may introduce social desirability bias and cannot objectively capture actual AI usage. Future studies could integrate behavioral log data (e.g., interaction frequency, duration, task type) with psychological measures to enhance ecological validity. Fourth, the study relied on purposive sampling through the authors’ social networks. Although this approach ensured that participants had prior experience with AI for IDLE, it may introduce self-selection bias, as individuals who are more motivated or more closely connected to the researchers could be overrepresented. Future studies should consider random or stratified sampling to enhance representativeness. Fifth, this study did not differentiate between types of AI tools (e.g., ChatGPT, DeepL, Grammarly) or usage tasks (e.g., writing, speaking, reading). Different tools and tasks may involve distinct psychological mechanisms. For instance, writing tasks may rely more on perceived usefulness, whereas conversational practice may depend more heavily on enjoyment. Future research should conduct task-specific analyses to capture these nuanced differences. Finally, English proficiency was assessed using a single self-reported item on a 5-point scale. Such single-item measures are vulnerable to self-report bias, as learners may misjudge their actual ability, and they cannot capture the multidimensional nature of language proficiency across reading, writing, speaking, and listening. As a result, this indicator may not accurately reflect objective linguistic competence. Future research should employ standardized assessments such as TOEFL, IELTS, or China’s College English Test (CET) to obtain more objective and fine-grained measures of English proficiency.

## 7. Conclusions

This study employed PLS-SEM and PNA to identify the key mechanisms underlying Chinese STEM undergraduates’ engagement in AI-IDLE. The results indicate that enjoyment was the most central driver, exerting both a strong direct effect on learning behavior and an indirect effect through enhanced focused immersion. Curiosity also significantly predicted learning behavior. Control contributed indirectly by promoting focused immersion and directly by stimulating usage intention, underscoring its distinctive value in autonomous learning contexts. In contrast, boredom had no significant effect on either focused immersion or learning behavior and was positioned at the periphery of the network. Perceived ease of use functioned as a foundational variable, significantly predicting perceived usefulness and multiple emotional experiences, and influencing learning behavior through several indirect pathways. Perceived usefulness directly predicted AI-IDLE in PLS-SEM, although its role was limited in PNA. Taken together, these findings suggest that STEM students’ AI-IDLE behavior is primarily driven by intrinsic hedonic motivations such as enjoyment, curiosity, and control, while instrumental evaluations play a supplementary role and boredom exerts minimal influence.

## Figures and Tables

**Table 1:** Demographic details of participants (*n* = 413).

Variables	Count	Percentage
Gender		
Male	212	51.3%
Female	201	48.7%
Age		
18–20	300	72.6%
21–25	79	19.1%
26–30	30	7.3%
>30	4	1%
Grade		
Undergraduate	303	73.4%
Graduate	68	16.5%
PhD candidate	42	10.2%

**Table 2:** Measurement model results (*n* = 413).

Construct	Item	Loading	AVE	CR	Cronbach’s α
Boredom	Bo1~Bo4	0.846~0.912	0.764	0.928	0.898
Control	CO1~Co4	0.887~0.904	0.797	0.922	0.873
Curiosity	CY1~CY3	0.842~0.942	0.810	0.945	0.923
Enjoyment	En1~En4	0.785~0.866	0.697	0.902	0.855
AI-IDLE	GI1~GI8	0.816~0.879	0.685	0.945	0.934
Focused immersion	IM1~IM4	0.766~0.858	0.672	0.891	0.839
Perceived ease of use	PE1~PE4	0.809~0.896	0.724	0.913	0.872
Perceived usefulness	PU1~PU5	0.776~0.932	0.703	0.922	0.894

*Note*: AVE = Average Variance Extracted; CR = composite reliability.

**Table 3:** Fornell–Larcker Criterion results (*n* = 413).

Construct	1	2	3	4	5	6	7	8
1. Boredom	**0.874**							
2. Control	−0.276	**0.893**						
3. Curiosity	0.045	0.146	**0.900**					
4. Enjoyment	−0.327	0.627	0.206	**0.835**				
5. Focused immersion	−0.142	0.558	0.317	0.541	**0.820**			
6. AI-IDLE	−0.176	0.591	0.265	0.717	0.570	**0.828**		
7. Perceived ease of use	−0.166	0.553	0.110	0.558	0.427	0.507	**0.851**	
8. Perceived usefulness	−0.190	0.564	0.072	0.645	0.481	0.571	0.765	**0.838**

*Note*: The bold values denote the AVE’s square root.

**Table 4:** Direct pathway results (*n* = 413).

Pathway	β	SD	t	*p*
Control variables				
Age → AI-IDLE	0.036	0.071	0.510	0.610
Gender → AI-IDLE	0.006	0.064	0.095	0.924
Grade → AI-IDLE	−0.020	0.072	0.280	0.780
Key constructs				
Boredom → Focused immersion	0.043	0.042	1.024	0.306
Boredom → AI-IDLE	0.051	0.040	1.286	0.199
Control → Focused immersion	0.360	0.056	6.459	0.000
Curiosity → Focused immersion	0.204	0.046	4.453	0.000
Curiosity → AI-IDLE	0.084	0.036	2.366	0.018
English proficiency → AI-IDLE	0.034	0.033	1.044	0.297
Enjoyment → Focused immersion	0.288	0.055	5.264	0.000
Enjoyment → AI-IDLE	0.507	0.057	8.847	0.000
Focused immersion → AI-IDLE	0.204	0.048	4.233	0.000
Perceived ease of use → Boredom	−0.166	0.056	2.967	0.003
Perceived ease of use → Control	0.553	0.044	12.490	0.000
Perceived ease of use → Curiosity	0.110	0.051	2.164	0.031
Perceived ease of use → Enjoyment	0.558	0.045	12.425	0.000
Perceived ease of use → Perceived usefulness	0.765	0.025	30.515	0.000
Perceived usefulness → AI-IDLE	0.145	0.063	2.304	0.021

**Table 5:** Indirect pathway results (*n* = 413).

Pathway	β	SD	t	Lower	Upper
Perceived ease of use → Perceived usefulness → AI-IDLE	0.110	0.049	2.259	0.017	0.207
Perceived ease of use → Boredom → Focused immersion → AI-IDLE	−0.002	0.002	0.911	−0.005	0.001
Perceived ease of use → Control → Focused immersion → AI-IDLE	0.041	0.012	3.353	0.019	0.066
Perceived ease of use → Enjoyment → AI-IDLE	0.284	0.038	7.421	0.210	0.357
Perceived ease of use → Curiosity → AI-IDLE	0.009	0.006	1.525	0.000	0.024
Perceived ease of use → Boredom → AI-IDLE	−0.007	0.007	0.967	−0.024	0.006
Perceived ease of use → Curiosity → Focused immersion → AI-IDLE	0.005	0.003	1.596	0.000	0.011
Perceived ease of use → Enjoyment → Focused immersion → AI-IDLE	0.033	0.010	3.248	0.015	0.055

## Data Availability

The data presented in this study are available on request from the corresponding author.

## Supplementary Materials

The following supporting information can be downloaded at: https://www.mdpi.com/article/10.3390/jintelligence14070120/s1, Original data.

## Abbreviations

The following abbreviations are used in this manuscript:

AI	Artificial intelligence
IDLE	Informal digital learning of English
HMSAM	Hedonic-Motivation System Adoption Model
PLS-SEM	Partial least squares structural equation modeling
PNA	Psychological network analysis
